# Effects of Selected Dietary Secondary Metabolites on Reactive Oxygen Species Production Caused by Iron(II) Autoxidation

**DOI:** 10.3390/molecules191220023

**Published:** 2014-12-01

**Authors:** Vladimir Chobot, Franz Hadacek, Lenka Kubicova, Maurizio Battino

**Affiliations:** 1 Division of Molecular Systems Biology, Department of Ecogenomics and Systems Biology, Faculty of Life Sciences, University of Vienna, Althanstrasse 14, A-1090 Vienna, Austria; E-Mail: lenka.kubicova@univie.ac.at; 2 Department of Plant Biochemistry, Albrecht-von-Haller Institut, Georg-August-Universität Göttingen, Justus-von-Liebig-Weg 11, D-37077 Göttingen, Germany; E-Mail: franz.hadacek@biologie.uni-goettingen.de

**Keywords:** iron(II) autoxidation, dietary antioxidants, polyphenols, superoxide, hydroxyl radical, free radicals, oxidative stress, aging, inflammation, chronic diseases

## Abstract

Iron is an essential co-factor for many enzymes that catalyze electron transfer reactions. It is well known that so-called “poorly liganded” iron can increase ROS concentrations and trigger oxidative stress that is capable of initiating apoptosis. Conversely, controlled ROS production has been recognized as an integral part of cellular signaling. Elevated ROS concentrations are associated with aging, inflammatory and degenerative diseases. Anti-aging properties have been attributed especially to antioxidant phenolic plant metabolites that represent food additives in our diet. Consequently, this study explores the effects of flavonoids (quercetin and rutin), several phenolic acids (caffeic, chlorogenic, and protocatechuic acid), and the alkaloid caffeine on iron(II) autoxidation and ROS production in comparison to the standard antioxidants ascorbic acid and Trolox. The iron(II) autoxidation assay was carried out in pH 6.0 (plant apoplast and inflamed human tissue) and 7.4 (cell cytoplasm and human blood plasma). The obtained results accentuate phenolic acids as the more specific antioxidants compared to ascorbic acid and Trolox. Flavonoid redox chemistry depends more on the chemical milieu, specifically on pH. *In vivo*, the presence of iron cannot be ruled out and “wrongly” or “poorly” complexed iron has been pointed out as causative agent of various age-related diseases.

## 1. Introduction

Iron ions catalyze various electron transfer reactions in plant and animal cells, mostly as enzymatic cofactors, but also in coordination complexes with low-molecular-weight metabolites or drugs. Uncontrolled one-electron transfers may lead to the production of reactive oxygen species (ROS), which are required for maintaining a redox homeodynamic equilibrium and signaling in low concentrations but can cause oxidative stress in higher concentrations [1,2,3,4]. High ROS concentrations have been identified as key factors in development of many degenerative processes contributing to aging [3,5,6]. The most dangerous ROS is the highly reactive hydroxyl radical (^•^OH) which is able to oxidize practically every molecule in the cell [7,8]. Hydroxyl radical arises by one electron reduction of hydrogen peroxide (Equation (1)):

H_2_O_2_ + Fe^II^ → ^−^OH + ^•^OH + Fe^III^ (1)

Reaction (1) is known as the Fenton reaction and iron(II) is an initiator of this reaction. Hydrogen peroxide can arise by a spontaneous or enzymatically catalyzed dismutation of superoxide anion radical (O_2_^•−^) (Equation (2)). The superoxide anion radical is a product of iron(II) autoxidation (Equation (3)) or accidental one-electron transfers in mitochondria or chloroplasts [9]:

2O_2_^•−^ + 2H^+^ → H_2_O_2_ + O_2_ (2)

O_2_ + Fe^II^ → O_2_^•−^ + Fe^III^ (3)

The stability of iron oxidation states depends on the pH of solution. Iron(II) is more stable in acidic solutions than in the alkaline ones where it becomes more easily oxidizable to iron(III) in the presence of molecular oxygen [10]. Biologically relevant pH values are 7.4 (cell cytoplasm and human blood plasma) and 6.0 (plant apoplast and inflamed human tissue) [11,12,13]. A pH lower than 6.0 can occur in special compartments and organs such as plant vacuoles, lysosomes or the human stomach [13,14,15].

Because of the presence of various reducing agents such as ascorbic acid, glutathione or NAD(P)H, iron occurs in cells mainly in its divalent state [16]. Redox properties of iron and its ability to produce ROS depend on the types of ligands which form complexes with iron. The ligands containing oxygen groups such as phenolic or carboxyl groups decrease the iron redox potential. Ligands with nitrogen or sulphur containing groups, such as for example in amines, the heterocycles imidazole and pyridine, and the thiol group of cysteine, by contrast, increase the redox potential of iron [10,17,18]. In addition, iron complexes with phenols, especially flavonoids, can catalyze superoxide anion dismutation similarly as superoxide dismutase (SOD) and enhance the rate of the redox reaction cascades, resulting in the Fenton reaction [19,20].

Plant polyphenols and some alkaloids such as caffeine occur ubiquitously in human diet. Daily uptake of these compounds may be in order of dozens or hundreds of milligrams because their concentrations in food plants can be very high [16,21,22,23], depending on the growth conditions [24,25,26]. The effects of plant metabolites and dietary supplements on human health have been a subject of discussions for a long time [4,27,28,29,30]. For example, the purine alkaloid caffeine is a constituent of very popular beverages, tea, coffee, and energy drinks. Recently, caffeine has been a topic of debates about beneficial effects of its chronic consumption on the withdrawing cognitive functions associated with aging [31]. Although the function of flavonoids, phenolic acids and alkaloids in the plant is still far from being fully understood, these compounds possessing redox and/or iron chelating properties have been attracting attention for possible preventing the onset of several degenerative diseases connected with aging [16,32,33,34,35,36]. 

The iron(II) autoxidation assay explores ROS formation dynamics connected with reduction of molecular oxygen that diffuses into aqueous reaction mixtures [37]. In the first step, molecular oxygen is reduced by iron(II) to superoxide anion radical (Equation (3)). The final product of a complex reaction cascade is the highly oxidative hydroxyl radical that attacks the detection molecule 2-deoxy-d-ribose. Its decomposition products can be quantified photometricaly after reaction with 2-thiobarbituric acid as thiobarbituric acid reactive species (TBARS). The added test compound can accelerate or slow down the rate of the 2-deoxy-d-ribose degradation by ROS scavenging and production and/or by acting as ligands in iron coordination complexes. The redox chemical properties of coordination complexes cannot be predicted easily because they depend both on the spin rates of the valence electrons and on steric factors, which affects the redox potentials of both central atom and ligands [10,17,18]. Many well-known antioxidants may show a pro-oxidant activity, either by directly reducing molecular oxygen to superoxide anion radical or by affecting Fe(II)/Fe(III) redox cycling [18,38,39,40].

**Figure 1:**
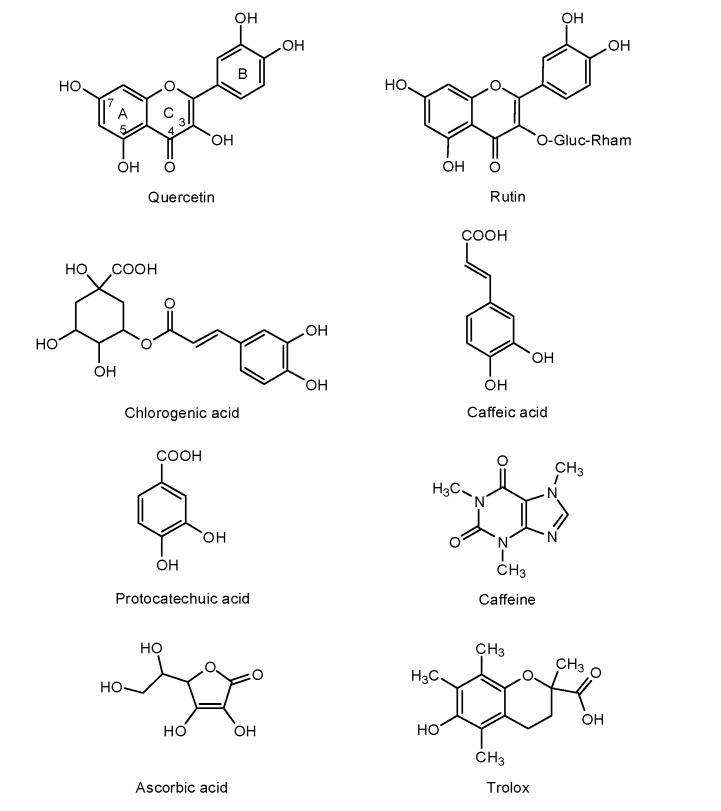
Structures of the tested compounds.

This paper explores the redox chemistry of selected substances with diverse structures, flavonoids, quercetin and rutin, phenolic acids, caffeic, chlorogenic, and protocatechuic acid, and the alkaloid caffeine, in comparision to standard antioxidants, ascorbic acid and a water soluble tocopherol analogue, Trolox (Figure 1), with a specific focus on iron(II) autoxidation and ROS formation in aqueous reaction mixtures at two different physiologically relevant pH values, 7.4 and 6.0.

## 2. Results and Discussion

**Figure 2:**
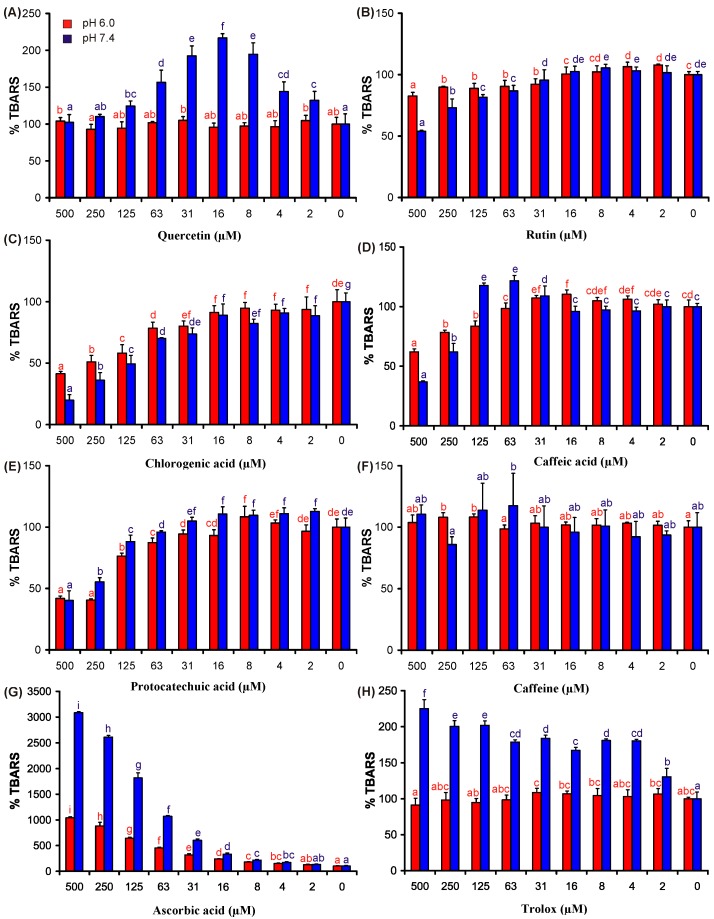
ROS generation in the Fe(II) autoxidation assay. Graphs (**A**)−(**H**) show activities of the tested compounds (A, quercetin; B, rutin; C, chlorogenic acid, D, caffeic acid; E, protocatechuic acid; F, caffeine; G, ascorbic acid; H, Trolox) in the assay. ROS were quantified as thiobarbituric acid reactive species (TBARS) arising from oxidative degradation of 2-deoxy-d-ribose. The results are presented as TBARS levels relative to the control (100% = TBARS of the control reaction mixture without test compound). The final concentration of Fe(II) in the reaction mixture was 10 µM. Error bars indicate standard deviation of three replicates; letters (a−i) indicate different levels of significance (95% Duncan), for details see Experimental Section.

The assay revealed interesting differences among the tested compounds in terms of their effects on iron(II) autoxidation and ROS-triggered 2-deoxy-d-ribose degradation (Figure 2). The pH effects were pronounced in some cases and negligible in others. 

The flavonoid quercetin was pro-oxidant in the concentration range 2−125 µM in pH 7.4 (Figure 2A). In the higher concentration range, 250−500 µM, quercetin’s ROS scavenging properties became more pronounced than its pro-oxidant activity. The dose-response curve showed an inverted U-shape form. At pH 6.0, however, the anti- and pro-oxidant activities disappeared. A possible reason for this effect can be a decreased dissociation of phenolic hydroxyl groups. Oxidation of phenolic hydroxyl groups depends on their dissociation and is more difficult to occur in acidic pH [41]. By contrast, rutin behaved as an antioxidant in both pHs (Figure 2B), the *o*-dihydroxyl group arrangement of ring B being most probably responsible. This points to the contribution of the non-substituted 3-hydroxyl group on ring C to the pro-oxidant activity of quercetin [42,43,44]. The pro-oxidant properties of quercetin at pH 7.4 depend on ability of quercetin and its semiquinone to reduce molecular oxygen to superoxide anion radical and hydrogen peroxide [38,45]. Quercetin is oxidized to semiquinone by superoxide anion radical, a reaction product of iron(II) autoxidation. Semiquinones of flavonoids with *o*-dihydroxyl or trihydroxyl groups are known as potent reducing agents which can reduce molecular oxygen to superoxide anion radical [38,45]. Superoxide anion radical dismutates or can be reduced to hydrogen peroxide by another quercetin molecule. These processes lead to a quick formation of hydrogen peroxide which enters in the Fenton reaction.

Both flavonoids are able to form coordination complexes with iron which can affect the studied 2-deoxy-d-ribose degradation in a multi-faceted fashion. The ability of complex formation of flavonoids with metals such as iron depends on the number of hydroxyl groups and their arrangement in the flavonoid molecule [46,47,48,49]. For example, flavonoids with *o*-dihydroxyl group on ring B as rutin and quercetin were identified as efficient iron chelators. The hydroxyl groups at positions 3 of ring C or 5 of ring A and 4-keto group of ring C also represent good coordination sites; the efficiency of the latter, however, is low in acidic environment [47,50]. Ligands with oxygen coordination atoms such as flavonoids do not prevent iron(II) autoxidation but stabilize iron in trivalent oxidation state by shifting the iron redox potential into the cathodic direction [10,16,17]. As a result of this effect, iron(II) is more easily oxidized to iron(III) but iron(III) back reduction to iron(II) is more difficult [17].

Perron *et al.* suggest that the polyphenols increasing the rate of iron(II) autoxidation protect DNA against oxidative damage. The authors propose a higher redox stability of iron(III) in iron(III)-flavonoid coordination complexes [46]. 

In contrast to the tested flavonoids, all phenolic acids, caffeic, chlorogenic and protocatechuic acid, proved to be efficient antioxidants in both tested pH environments (Figure 2C–E). Their activity was slightly more pronounced at the alkaline pH than in the acidic reaction mixture due to better dissociation and increased electron density of the carboxylate. Chlorogenic acid was the most active antioxidant of all tested compounds. The effect of caffeic acid was comparable to the activity of protocatechuic acid. However, this contradicts reports suggesting that ROS scavenging activity of hydroxycinnamic acids is higher than that of corresponding hydroxybenzoic acids because of the extended electron delocalization [44,51] and possible addition of hydroxyl radicals to the vinyl group of the cinnamic acid derivatives [52,53]. This points to the fact that not only ROS scavenging, but also inhibition of iron catalytic ability and iron redox cycling in coordination complexes contribute to the antioxidant properties of phenolic acids [16]. Acids with *o*-dihydroxyl groups have been pointed out as potent iron chelators, chlorogenic acid > caffeic acid > protocatechuic acid [54], by forming stable coordination complexes with trivalent iron. Caffeic and protocatechuic acids efficiently promoted iron(II) autoxidation in the ferrozine assay that was carried out in HEPES buffer [55]. Conversely, other reports suggested that chlorogenic and caffeic acid were able to reduce iron(III) back to reactive iron(II) in the iron-acid complexes by one electron intermolecular transfer [56]. These reactions were carried out in acidic environment where iron(III) is more reactive than in neutral or alkaline solutions [10]. At pH 7.4, Chvatalova *et al.* reported only slow reduction of iron(III) in chlorogenic or caffeic acid complexes [55]. None of the tested phenolic acids showed detectable pro-oxidant activity in the iron(II) autoxidation assay. Consequently, our results concur with those reported by Chvatalova *et al.* [55]. 

The alkaloid caffeine showed no antioxidant activity in the range of the tested concentrations (Figure 2F), although caffeine is able to form adducts with hydroxyl radical, predominantly at the site C8 [57]. However, caffeine has been identified as a bad scavenger of hydroperoxyl radical, a protonated form of superoxide anion radical [57,58], and as a weak iron chelating agent [59]. The lack of protective effects of caffeine against oxidative degradation of 2-deoxy-d-ribose can be explained by lower caffeine reactivity towards hydroxyl radical compared to 2-deoxy-d-ribose as well as by insufficient inhibition of iron catalytic properties by iron−caffeine coordination complexes.

The standard antioxidants, ascorbic acid and Trolox, did not prevent iron(II) autoxidation and ROS formation in both of the tested pH reaction conditions (Figure 2G,H). They apparently increased TBARS concentrations in the reaction solutions of pH 7.4. The evident pro-oxidant activities suggest a significant promotion of iron(II)/iron(III) redox cycling if iron is liganded by ascorbic acid or Trolox [18,60]. In contrast, ascorbic acid and Trolox showed antioxidant activity in other assays using various hydrophilic and lipophilic detection molecules [61]. It can be explained by different reaction rates of ROS with 2-deoxy-d-ribose compared to other detection molecules [62]. In the acidic reaction solutions, ascorbic acid was highly pro-oxidant whereas Trolox showed no significant activity. Our results agree with reported pro-oxidant effects of ascorbate in cancer chemotherapy [63]. 

## 3. Experimental Section

### 3.1. Chemicals

All chemicals used were purchased from Sigma-Aldrich (Schnelldorf, Germany). Water used was of Milli-Q quality.

### 3.2. Fe(II) Autoxidation Assay

The sample was dissolved in aqueous KH_2_PO_4_/KOH buffer solution (30 mM, pH 7.4) and diluted serially; to this solution (125 μL), a 52 mM 2-deoxy-d-ribose solution (25 μL) in the same buffer system, the buffer (50 µL), and degassed aqueous FeSO_4_ solution (50 μL, 50 μM) were added. The final concentrations of the tested compounds were 2–500 µM. Blanks contained the full reaction mixtures except for 2-deoxy-d-ribose. Standard 1.5 mL sample vials (La-Pha-Pack, Werner Reifferscheidt GmbH, Langerwehe, Germany) were used as reaction vials. The mixture was incubated at 27 °C for 16 h. Thereafter, 250 μL of 1.0% thiobarbituric acid dissolved in 3% trichloroacetic acid (w/v) was added to each vial to detect TBARS. The vials were heated in a water bath at 80 °C for 30 min. The reaction was stopped by transferring the vials into an ice water bath for 3 min. To extract the TBARS, 600 μL of 1-butanol was added, and the mixture was rigorously vortexed. The butanol layers of the vials, each 350 μL, were pipetted into flat bottomed 96 well plates (Greiner, Kremsmünster, Austria), and the absorbance was determined with a microplate reader (Tecan Infinite M200, Männedorf, Switzerland) at 532 nm. Experiments were performed in triplicate. Reaction mixtures lacking the test compound served as the positive control (100% TBARS). The phosphate buffer and water, which were used as solvents for the tested substances or FeSO_4_, were degassed by argon for 10 min at least. The assay was performed in aqueous solutions and phosphate buffer because organic buffers and solvents react with hydroxyl radicals [64].

### 3.3. Statistical Analysis

Statgraphics Centurion XVI (Statistical Graphics Corp., Rockville, MD, USA) was used to perform analyses of variance (ANOVA) employing 95% Duncan’s multiple range *post hoc* test.

## 4. Conclusions

In oxidative stress conditions, iron may be released from its coordination complexes with proteins or other ligands and can catalyze further production of ROS. The *in vitro* results presented here suggest that secondary plant metabolites, for example phenolic acids, possess considerable potential to protect molecular cell structures against the oxidative stress even at lower pH which accompanies inflammation processes often associated with degenerative diseases and aging. At cytosolic pH, plant metabolites may increase ROS concentration by reducing molecular oxygen to ROS thereby affecting the rates of chemical reactions that are involved in iron redox cycling. The final activity depends on ratio between pro-oxidant and antioxidant processes. Generally, as the presented results demonstrate, reducing activity does not always correlate with capacity to inhibit oxidation [65]. A good example is ascorbic acid, for which the strong pro-oxidative effect that was evident in the assay concurs with its application in cancer chemotherapy. Redox active plant metabolites can contribute to maintaining the redox homeodynamic equilibrium in the cells [4,8,66]. Redox homeodynamic equlibrium implies that certain levels of ROS and oxidative products of some biomolecules such as lipids have to be present as signal molecules for regulation of metabolism [4]. Dietary redox active metabolites have thus potential to exert health-beneficial effects in certain patients, but also none or even detrimental ones in others. The reason is the complexity of involved chemistry revealed by the assay that was performed for this study. 
